# Reporting Quality of AI Intervention in Randomized Controlled Trials in Primary Care: Systematic Review and Meta-Epidemiological Study

**DOI:** 10.2196/56774

**Published:** 2025-02-25

**Authors:** Jinjia Zhong, Ting Zhu, Yafang Huang

**Affiliations:** 1 School of General Practice and Continuing Education Capital Medical University Beijing China

**Keywords:** artificial intelligence, randomized controlled trial, reporting quality, primary care, meta-epidemiological study

## Abstract

**Background:**

The surge in artificial intelligence (AI) interventions in primary care trials lacks a study on reporting quality.

**Objective:**

This study aimed to systematically evaluate the reporting quality of both published randomized controlled trials (RCTs) and protocols for RCTs that investigated AI interventions in primary care.

**Methods:**

PubMed, Embase, Cochrane Library, MEDLINE, Web of Science, and CINAHL databases were searched for RCTs and protocols on AI interventions in primary care until November 2024. Eligible studies were published RCTs or full protocols for RCTs exploring AI interventions in primary care. The reporting quality was assessed using CONSORT-AI (Consolidated Standards of Reporting Trials–Artificial Intelligence) and SPIRIT-AI (Standard Protocol Items: Recommendations for Interventional Trials–Artificial Intelligence) checklists, focusing on AI intervention–related items.

**Results:**

A total of 11,711 records were identified. In total, 19 published RCTs and 21 RCT protocols for 35 trials were included. The overall proportion of adequately reported items was 65% (172/266; 95% CI 59%-70%) and 68% (214/315; 95% CI 62%-73%) for RCTs and protocols, respectively. The percentage of RCTs and protocols that reported a specific item ranged from 11% (2/19) to 100% (19/19) and from 10% (2/21) to 100% (21/21), respectively. The reporting of both RCTs and protocols exhibited similar characteristics and trends. They both lack transparency and completeness, which can be summarized in three aspects: without providing adequate information regarding the input data, without mentioning the methods for identifying and analyzing performance errors, and without stating whether and how the AI intervention and its code can be accessed.

**Conclusions:**

The reporting quality could be improved in both RCTs and protocols. This study helps promote the transparent and complete reporting of trials with AI interventions in primary care.

## Introduction

Primary care provides a large health care delivery platform for primary care physicians to offer person-centered services for a vast patient population [1]. The practice of primary care generates substantial digital data that can be leveraged for primary care research [2,3]. The growing resources of big data, mainly consisting of electronic health records, electronic medical data, patient self-reported data, and wearable device data, have paved the way for the advancement of artificial intelligence (AI) in deep machine learning (ML) technology and natural language processing to explore AI- or ML-driven clinical tools dedicated to the improvement of clinical decision-making in the disease screening, diagnosis, prognosis, and management [4-6]. For example, several studies in primary care have developed the prediction tool for patients presenting with acute cough, the prediction score for head and neck cancer referrals, and the prediction model for asthma exacerbation [7-9]. Nevertheless, in practice, a significant proportion of these tools have not undergone validation through a robust clinical trial, although the majority of these studies use performance indicators to showcase their superiority [10,11].

Randomized controlled trials (RCTs) are widely recognized as the gold standard for evaluating interventions [12]. Many clinical studies that investigate AI- or ML-driven clinical tools as a clinical intervention conduct a confirmatory RCT, aiming to demonstrate the clinical significance [13-15]. Transparency and explainability are essential for the widespread integration of AI systems into clinical practice, as an inaccurate prediction could result in severe consequences [16]. Given the inherent complexity of AI interventions, it is critical for RCT reporting to be comprehensive and transparent. Adhering to reporting guidelines for AI in clinical trials and transparently reporting AI interventions in RCTs are important steps toward improving research quality, fostering scientific discourse, and establishing more reliable foundations for clinical practice and decision-making [17-19].

The CONSORT-AI (Consolidated Standards of Reporting Trials–Artificial Intelligence) extension [17] and SPIRIT-AI (Standard Protocol Items: Recommendations for Interventional Trials–Artificial Intelligence) extension [18] are newly developed reporting guidelines specifically focusing on AI intervention in trials. CONSORT-AI focuses more on trial results and reporting completeness, while SPIRIT-AI emphasizes pretrial elements, such as trial design and ethical considerations. The relative items in the guidelines should be transparently and completely reported, and qualified reporting is essential for independently evaluating and replicating the trial. However, there is no systematic review and critical appraisal of RCTs for examining AI interventions in primary care, leaving the clarity of their reporting quality uncertain.

This systematic review and meta-epidemiological study aims to evaluate the reporting quality of AI interventions in published RCTs and protocols for RCTs in primary care. The assessment was based on the CONSORT-AI extension and SPIRIT-AI extension guidelines.

## Methods

### Study Design

This study was reported in accordance with the guidelines for reporting meta-epidemiological methodology research [20] and PRISMA (Preferred Reporting Items for Systematic Reviews and Meta-Analysis) reporting guidelines (Multimedia Appendix 1). The protocol for this study has been registered on PROSPERO (ID: CRD42023427694).

### Search Strategy

The searches of PubMed, Embase, and Cochrane Library were conducted up to November 30, 2024. To ensure a comprehensive literature search, we further referenced a previously published study strategy [3], ensuring the completeness and accuracy of the process of collecting RCTs. This further search was applied across MEDLINE, Web of Science, and CINAHL. There were no restrictions on the year of publication or language. The detailed search strategies are listed in Multimedia Appendix 2.

### Study Selection

#### Overview

To be included in this study, articles had to meet the following criteria: (1) RCTs or protocols for RCTs that used AI interventions or AI-assisted tools to guide a randomized intervention and (2) studies that belonged to primary care research. Based on the 1996 report of the US Institute of Medicine, primary care was defined as “the provision of integrated, accessible health care services by clinicians who are accountable for addressing a large majority of personal health care needs, developing a sustained partnership with patients, and practicing in the context of family and community” [21]. Primary care research was defined as “Research done in a primary care context” [22]. Only published RCTs and published, full RCT protocols were included. We excluded (1) studies with abstracts only; (2) commentaries, letters, editorials, or reviews; and (3) animals or preclinical studies.

#### Stage 1: Title and Abstract Screening

The retrieved records were imported into EndNote 20 (Clarivate). Two independent reviewers (JZ and TZ) conducted the screening of titles and abstracts to identify studies potentially meeting the inclusion criteria. Any discrepancies were resolved through discussion.

#### Stage 2: Full-Text Screening

Using EndNote 20, the reviewers (JZ and TZ) independently assessed the full-text articles identified at stage 1 for eligibility. Any discrepancies were discussed and resolved by consulting the third researcher (YH), and consensus was reached through discussion. Studies meeting the eligibility criteria were included for data extraction.

### Data Extraction Process and Data Items

Two researchers (JZ and TZ) independently extracted data from each article by using a standardized data extraction form. Discrepancies in data extraction were discussed between JZ and TZ and the remaining conflicts were resolved by YH.

Data were collected on (1) trial name, (2) trial registration identifier assigned to clinical trials registered in the database, (3) first author’s name, (4) published article name, (5) journal name, (6) publication year, (7) research topic, (8) study design (sample size, blinding, and type of comparator arm), (9) primary outcome, (10) classification of primary outcomes by result status (positive or negative), (11) type of AI model (large language model [LLM], ML, deep learning, clinical decision support system [CDSS], or risk prediction model), and (12) deployment context (clinician-assisted decision support, fully automated diagnostic systems, or patient self-management tools).

The reporting quality of included studies was assessed using the CONSORT-AI and SPIRIT-AI checklists. Each checklist consists of two components: (1) the original CONSORT 2010/SPIRIT 2013 items and (2) the AI-specific elaborations and extension items. This study focused on evaluating the AI-related reporting quality. Only items that are separately elaborated or extended in the CONSORT-AI (14 items) and SPIRIT-AI (15 items) checklists were appraised. These items ensure that critical aspects of AI-supported interventions such as input-data setting are transparently and completely reported. Each item was assessed in all included published articles, as “Yes” or “No.”

### Outcomes

The primary outcomes were as follows: (1) the proportion of adequately reported items, calculated by dividing the number of adequately reported items by the total number of items in each article—high proportions would indicate high reproducibility and quality of the RCTs and protocols for RCTs—and (2) the percentage of articles adequately reporting each item, calculated by dividing the number of articles that adequately reported the item by the total number of articles evaluated for that particular item. Independent analysis was conducted for CONSORT-AI (14 items) and SPIRIT-AI (15 items) checklists based on the primary outcomes described above. Since CONSORT-AI focuses on reporting completed trials and SPIRIT-AI on trial protocol standards, analyzing them separately ensures more precise adherence to each guideline. The secondary outcomes focused on CONSORT-AI included (1) the association between primary outcome status and the reporting quality of each CONSORT-AI item, and (2) factors influencing the percentage of adequately reported items in CONSORT-AI.

### Data Synthesis and Analysis

Descriptive statistical methods were used and presented as frequencies, median percentages, and IQRs. The proportion of adequately reported items and the percentage of articles adequately reporting each specific item was reported, with its corresponding 95% CI calculated using the Clopper-Person method. The analyses were performed using Microsoft Excel 2021 and OriginPro 2022 (OriginLab Corporation). The association between primary outcome result status (positive or negative) and the reporting quality of each item was examined using the Fisher exact test. The percentage of adequately reported items was compared across RCT characteristics such as type of disease using the Mann-Whitney *U* test. To assess the main effects and interaction effects between the primary outcome result status and various RCT characteristics on the percentage of adequately reported items, the Scheirer-Ray-Hare test was used. All *P* values were derived from 2-sided tests, with significance defined as *P*<.05 (R, version 4.4.2; R Foundation for Statistical Computing).

## Results

### Overview

**Figure 1:**
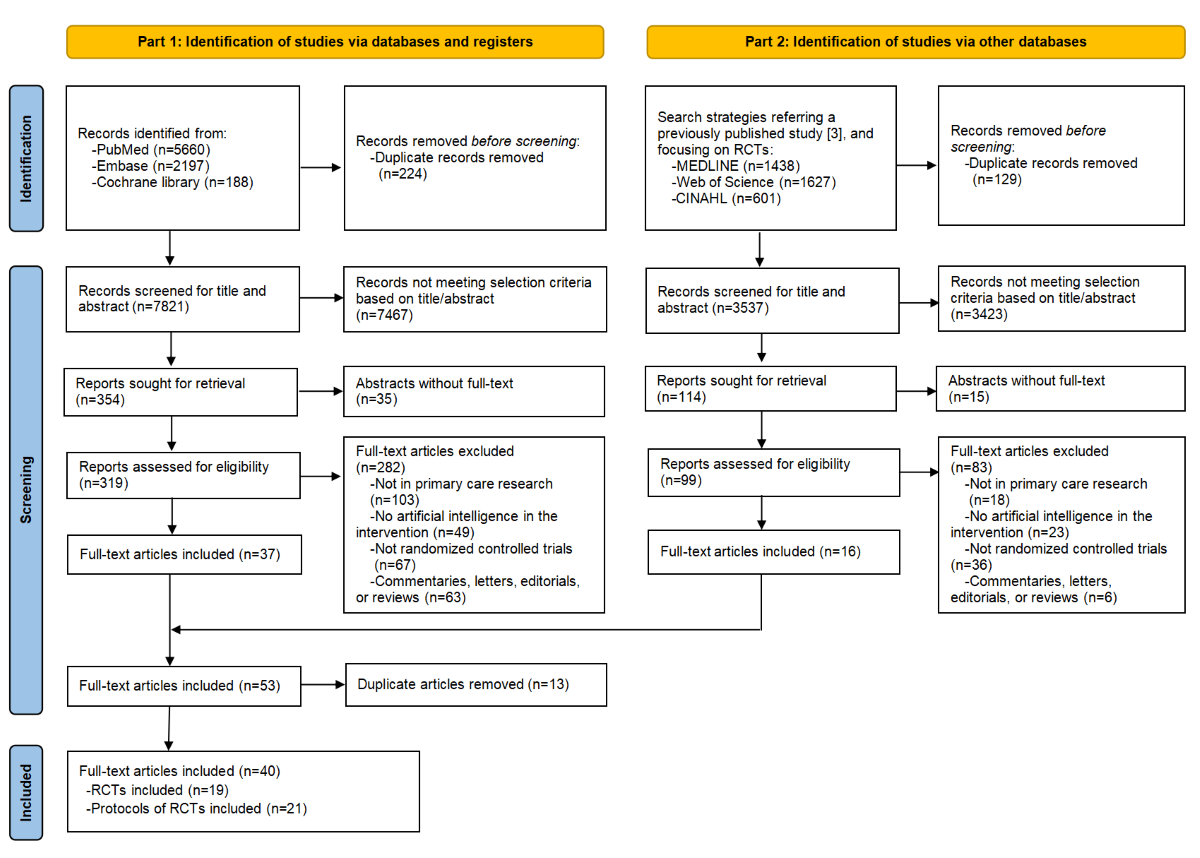
PRISMA (Preferred Reporting Items for Systematic Reviews and Meta-Analysis) flow diagram of the selection procedure. RCT: randomized controlled trial.

The search strategies retrieved a total of 11,711 records. After screening, 40 articles finally met the inclusion criteria. These 40 full-text articles corresponded to 35 trials, including 19 RCTs and 21 protocols for RCTs (Figure 1 and Multimedia Appendix 3 [23-62]).

### Characteristics of Included Articles

Table 1 summarizes the characteristics of the included articles. Among the 19 RCTs, 8 (42%) used LLMs, 10 (53%) were conducted in the context of clinician-assisted decision support, and 11 (58%) reported positive primary outcome results. In total, 21% (4/19) focused on cardiovascular topics. The median sample size for these RCTs was 335 (IQR 133-487) participants. Among the 21 protocols, 6 (29%) incorporated LLMs, 11 (52%) involved clinician-assisted decision support, and 6 (29%) focused on cardiovascular topics (Table 1).

**Table 1:** Characteristics of included published randomized controlled trials (RCTs) and protocols for RCTs.

Characteristics			Randomized controlled trials (n=19)		Protocols (n=21)		Overall (n=40)
Reported items, median (range)			9 (7-14)		10 (7-13)		9 (7-14)
**Publication year, n (%)**							
	2024	7 (37)		3 (14)		10 (25)	
	2023	3 (16)		2 (10)		5 (13)	
	2022	6 (32)		9 (43)		15 (38)	
	Pre-2021	3 (16)		7 (33)		10 (25)	
Sample size, median (IQR)			335 (133-487)		—^a^		—
**Blinding, n (%)**							
	Single blinded	6 (32)		2 (10)		8 (20)	
	Double-blinded	1 (5)		3 (14)		4 (10)	
	Open label	12 (63)		16 (76)		28 (70)	
**Type of comparator arm, n (%)**							
	Usual care	18 (95)		17 (81)		35 (88)	
	No treatment	1 (5)		3 (14)		4 (10)	
	Delay intervention	0 (0)		1 (5)		1 (3)	
**Type of artificial intelligence model, n (%)**							
	Large language model	8 (42)		6 (29)		14 (35)	
	Machine learning	2 (11)		4 (19)		6 (15)	
	Deep learning	6 (32)		6 (29)		12 (30)	
	Clinical decision support system	2 (11)		3 (14)		5 (13)	
	Risk prediction model	1 (5)		2 (10)		3 (8)	
**Deployment context, n (%)**							
	Clinician-assisted decision support	10 (53)		11 (52)		21 (53)	
	Fully automated diagnostic systems	3 (16)		1 (5)		4 (10)	
	Patient self-management tools	6 (32)		9 (43)		15 (38)	
**Classification of primary outcomes by result status, n (%)**							
	Positive	11 (58)		—		—	
	Negative	8 (42)		—		—	
**Disease or topic, n (%)**							
	Cardiovascular disease	4 (21)		6 (29)		10 (25)	
	Oncology	2 (11)		3 (14)		5 (13)	
	Pain management	4 (21)		1 (5)		5 (13)	
	Endocrine disorders	1 (5)		2 (10)		3 (8)	
	Respiratory diseases	2 (11)		1 (5)		3 (8)	
	Digestive disorders	2 (11)		0 (0)		2 (5)	
	Mental illness	1 (5)		2 (10)		3 (8)	
	Others	3 (16)^b^		6 (29)^c^		9 (23)	

^a^Not applicable.

^b^Prescription management, diagnostic reasoning, and quitting smoking.

^c^Auxiliary diagnosis, prescription management, retinal disease, quit smoking, and drug overdose.

### Proportion of Adequately Reported Items

In the 19 RCTs included in this review, the overall proportion of adequately reported items was 65% (172/266; 95% CI 59%-70%). Only 2 RCTs had more than 90% of items reported adequately. In the 21 protocols for RCTs, the overall proportion of adequately reported items was 68% (214/315; 95% CI 62%-73%). Two protocols of RCTs had more than 85% of items reported adequately (Multimedia Appendix 3).

### Percentage of Articles Adequately Reporting Each Specific Item

**Figure 2:**
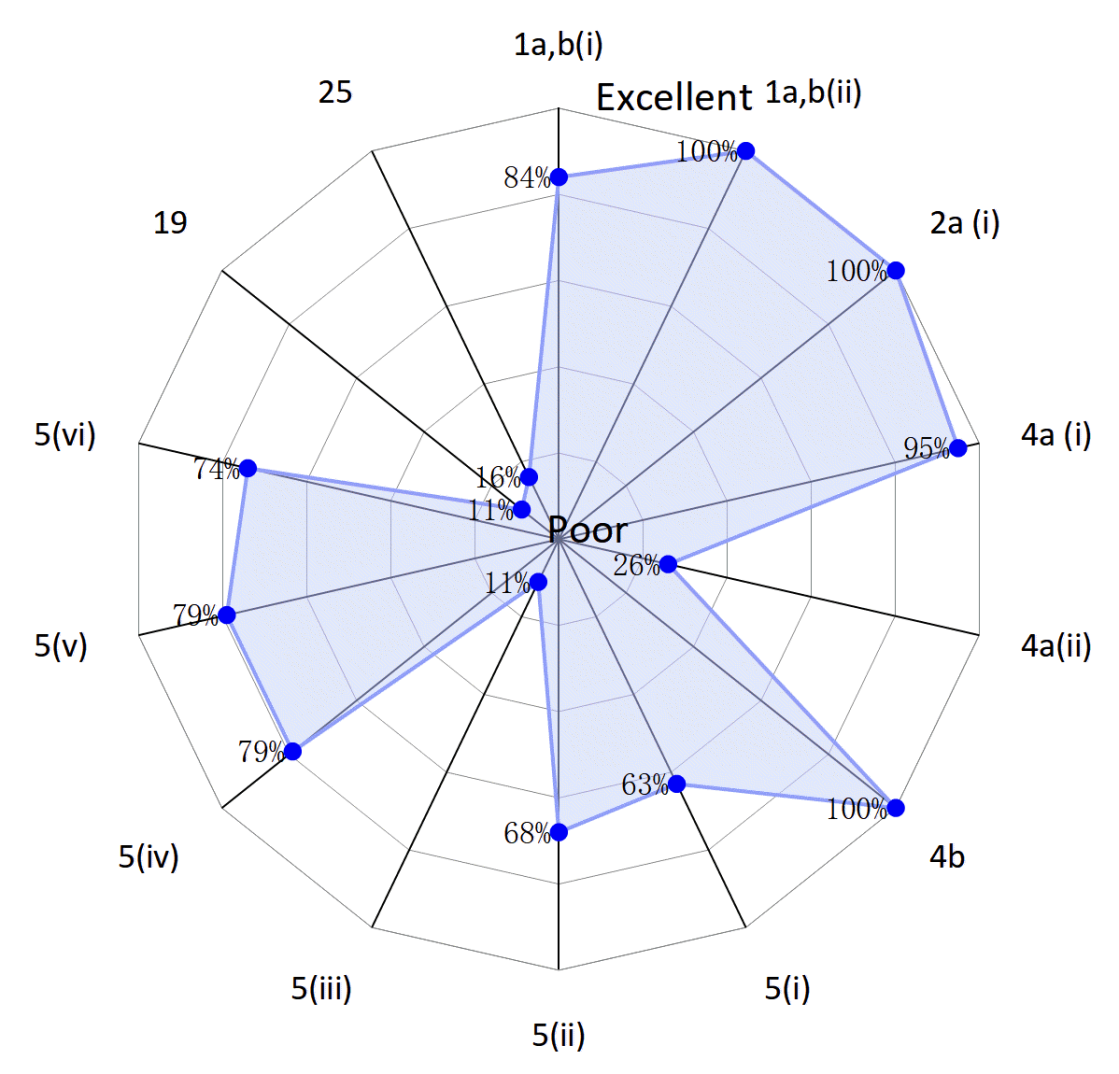
Radar chart to show the adequately reported percentage for each CONSORT-AI (Consolidated Standards of Reporting Trials–Artificial Intelligence) item.

The complete list of the 14 items of the CONSORT-AI extension is shown in Table 2. The percentage of RCTs that reported a specific item ranged from 11% to 100%. The best-reported sections were the title and abstract [item 1a and item b(ii)], background and objective [item 2a(i)], and participants (item 4b), all being reported in 100% (19/19) of RCTs. The poorly reported sections were intervention [item 5(i), item 5(ii), and item 5(iii)], participants [item 4a(ii)], harms (item 19), and funding (item 25). Among these poorly reported items, 4a(ii), 5(ii), and 5(iii) were related to providing the information for input data (Figure 2 and Table 2). The reporting quality increases as it approaches the outer edges of the radar chart—1a, b(i), 1a,b(ii): title; 2a(i): background and objectives; 4a(i), 4a(ii), 4b: participants; 5(i)-5(iv): intervention; 19: harms; and 25: funding.

**Table 2:** Adherence to CONSORT-AI (Consolidated Standards of Reporting Trials–Artificial Intelligence) extension items.

Item	Item description	Total (n=19), n (%; 95%CI)
CONSORT-AI 1a Elaboration	Indicate that the intervention involves artificial intelligence or machine learning in the title and abstract and specify the type of model.	16 (84; 60-97)
CONSORT-AI 1b(ii) Elaboration	State the intended use of the AI^a^ intervention within the trial in the title and abstract.	19 (100; 82-100)
CONSORT-AI 2a(i) Extension	Explain the intended use of the AI intervention in the context of the clinical pathway, including its purpose and its intended users (eg, health care professionals, patients, public).	19 (100; 82-100)
CONSORT-AI 4a(i) Elaboration	State the inclusion and exclusion criteria at the level of participants	18 (95; 74-100)
CONSORT-AI 4a(ii) Extension	State the inclusion and exclusion criteria at the level of the input data.	5 (26; 9-51)
CONSORT-AI 4b Extension	Describe how the AI intervention was integrated into the trial setting, including any onsite or offsite requirements.	19 (100; 82-100)
CONSORT-AI 5(i) Extension	State which version of the AI algorithm was used.	12 (63; 38-84)
CONSORT-AI 5(ii) Extension	Describe how the input data were acquired and selected for the AI intervention.	13 (68; 43-87)
CONSORT-AI 5(iii) Extension	Describe how poor quality or unavailable input data were assessed and handled.	2 (11; 1-33)
CONSORT-AI 5(iv) Extension	Specify whether there was human-AI interaction in the handling of the input data, and what level of expertise was required of users.	15 (79; 54-94)
CONSORT-AI 5(v) Extension	Specify the output of the AI intervention.	15 (79; 54-94)
CONSORT-AI 5(vi) Extension	Explain how the AI intervention’s outputs contributed to decision-making or other elements of clinical practice.	14 (74; 49-91)
CONSORT-AI 19 Extension	Describe results of any analysis of performance errors and how errors were identified, where applicable. If no such analysis was planned or done, justify why not.	2 (11; 1-33)
CONSORT-AI 25 Extension	State whether and how the AI intervention and/or its code can be accessed, including any restrictions to access or re-use.	3 (16; 3-40)

^a^AI: artificial intelligence.

**Figure 3:**
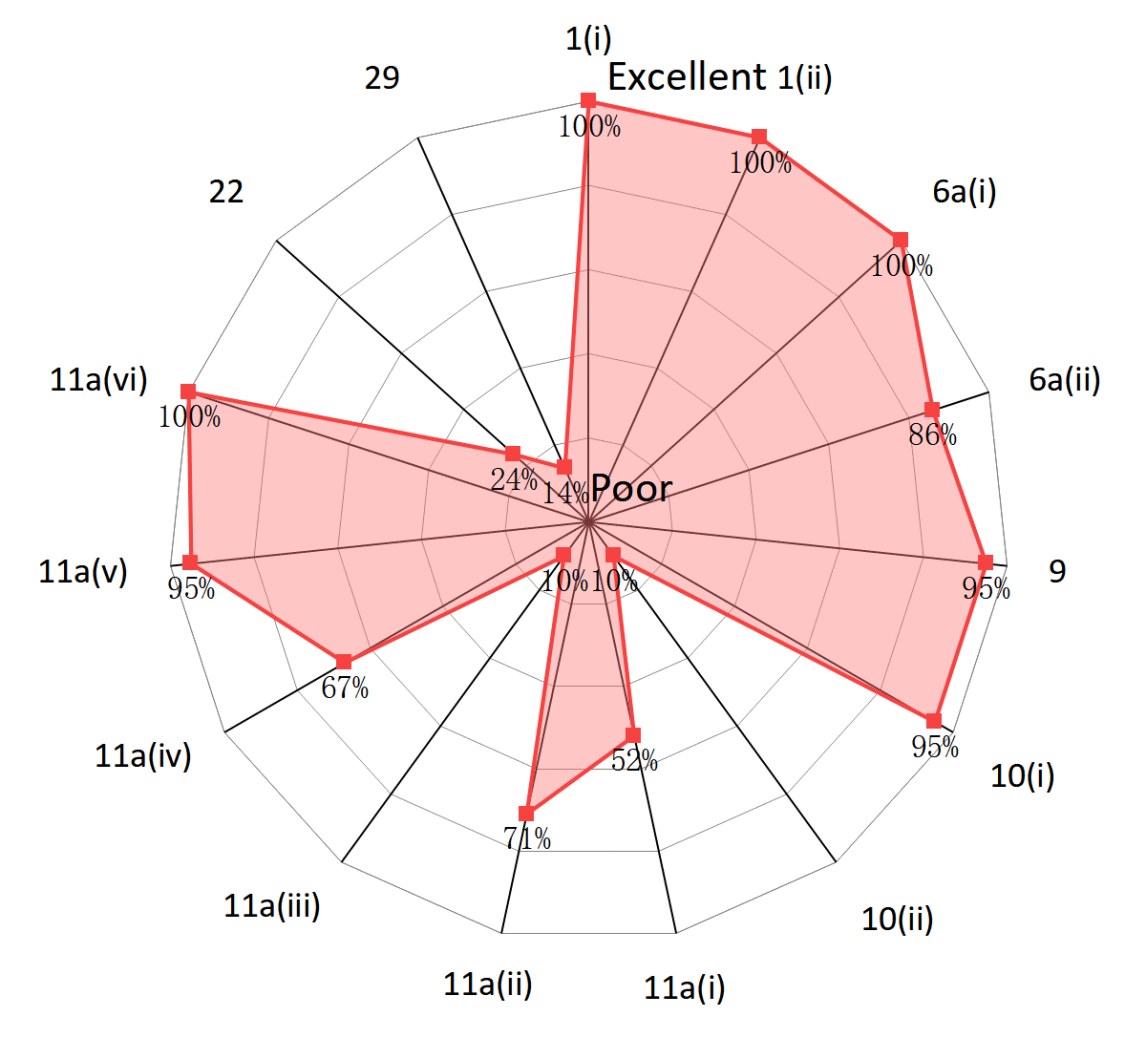
Radar chart to show the adequately reported percentage for each SPIRIT-AI (Standard Protocol Items: Recommendations for Interventional Trials–Artificial Intelligence) item.

The complete list of the 15 items of the SPIRIT-AI extension is shown in Table 3. The percentage of protocols for RCTs that reported a specific item ranged from 10% to 100%. The best-reported sections were title and abstract [item 1(i) and item 1(ii)], background and rationale [item 6a(i)], and intervention [item 11a(vi)] all being reported in 100% of protocols. The poorly reported sections were intervention [item 11a(i), item 11a(iii), and item 11a(iv)], eligibility [item 10(ii)], harms (item 22), and access to data (item 29). Among these poorly reported items, 10(ii), 11a(iii), and 11a(iv) were related to providing the information for input data (Figure 3 and Table 3). The reporting quality increases as it approaches the outer edges of the radar chart—1(i), 1(ii): title; 6a(i), 6a(ii): background and rationale; 9: study setting; 10(i), 10(ii): eligibility criteria; 11a(i)-11a(vi): interventions; 22: harms; and 29: access to data.

**Table 3:** Adherence to SPIRIT-AI (Standard Protocol Items: Recommendations for Interventional Trials—Artificial Intelligence) extension items.

Item	Item description	Total (n=21), n (%; 95% CI)
SPIRIT-AI 1(i) Elaboration	Indicate that the intervention involves artificial intelligence or machine learning and specify the type of model.	21 (100; 84-100)
SPIRIT-AI 1(ii) Elaboration	Specify the intended use of the AI^a^ intervention.	21 (100; 84-100)
SPIRIT-AI 6a(i) Extension	Explain the intended use of the AI intervention in the context of the clinical pathway, including its purpose and its intended users (eg, health care professionals, patients, public).	21 (100; 84-100)
SPIRIT-AI 6a(ii) Extension	Describe any pre-existing evidence for the AI intervention.	18 (86; 64-97)
SPIRIT-AI 9 Extension	Describe the onsite and offsite requirements needed to integrate the AI intervention into the trial setting.	20 (95; 76-100)
SPIRIT-AI 10(i) Elaboration	State the inclusion and exclusion criteria at the level of participants.	20 (95; 76-100)
SPIRIT-AI 10(ii) Extension	State the inclusion and exclusion criteria at the level of the input data.	2 (10; 1-30)
SPIRIT-AI 11a(i) Extension	State which version of the AI algorithm will be used.	11 (52; 30-74)
SPIRIT-AI 11a(ii) Extension	Specify the procedure for acquiring and selecting the input data for the AI intervention.	15 (71; 48-89)
SPIRIT-AI 11a(iii) Extension	Specify the procedure for assessing and handling poor quality or unavailable input data.	2 (10; 1-30)
SPIRIT-AI 11a(iv) Extension	Specify whether there is human-AI interaction in the handling of the input data, and what level of expertise is required for users.	14 (67; 43-85)
SPIRIT-AI 11a(v) Extension	Specify the output of the AI intervention.	20 (95; 76-100)
SPIRIT-AI 11a(vi) Extension	Explain the procedure for how the AI intervention’s output will contribute to decision-making or other elements of clinical practice.	21 (100; 84-100)
SPIRIT-AI 22 Extension	Specify any plans to identify and analyze performance errors. If there are no plans for this, explain why not.	5 (24; 8-47)
SPIRIT-AI 29 Extension	State whether and how the AI intervention and/or its code can be accessed, including any restrictions to access or re-use.	3 (14; 3-36)

^a^AI: artificial intelligence.

### Association Between Primary Outcome Result Status and the Reporting Quality of Each Item

Multimedia Appendix 4 shows the association between the primary outcome result status in RCTs and the reporting quality of each item. Fisher exact test results indicate no significant association between positive outcomes and higher reporting quality (all *P*>.05).

### Factors Associated With the Percentage of Adequately Reported Items

To further explore potential differences resulting from RCT characteristics, we compared the percentage of adequately reported items by year of publication, blinding, type of AI model, deployment context, primary outcome result status, and disease type (Multimedia Appendix 5). Trials of cardiovascular disease showed a higher percentage of adequately reported items (*P*=.02). The Scheirer-Ray-Hare test did not detect statistical significance for the interaction effects between primary outcome result status and various RCT characteristics on the percentage of adequately reported items (all *P*>.05; Multimedia Appendix 5).

## Discussion

### Principal Results

In this study, we assessed the RCT reporting quality in published 19 RCTs and 21 RCT protocols involved in 35 trials, with 5 trials having both published RCTs and corresponding protocols. This study found the reporting quality in both RCTs and protocols for RCTs of AI intervention could be improved in 3 aspects. The first aspect concerns providing enough information for the input data, which was detailed in CONSORT-AI guidelines [items 4a(ii), 5(ii), and 5(iii)] and SPIRIT-AI guidelines [items 10(ii), 11a(iii), and 11a(iv)]. The second aspect involves the lack of information on how to identify and analyze performance errors, which was demanded by the harm section of CONSORT-AI guidelines (item 19) and SPIRIT-AI guidelines (item 22), respectively. The third aspect involves neglecting to specify whether and how the AI intervention or its code can be accessed, which was needed in CONSORT-AI guidelines (item 25) and SPIRIT-AI guidelines (item 29), respectively. Based on the evaluation of the reporting of 35 trials, our findings revealed a consistent lack of information across these three aspects across both protocols and RCTs, highlighting a widespread gap in adhering to the reporting standards recommended by these guidelines.

### Strength and Limitations

This is the first study focused on the field of AI application in primary care, providing a first systematic review into the existing landscape of RCTs using AI interventions within primary care. This review not only included RCTs of AI interventions in primary care but also covered protocols, providing a more comprehensive survey in this field.

This study has 2 limitations. First, the majority of studies were conducted in the United States, which may lead to a lack of representation for other countries. Second, this study assessed the reporting quality of AI intervention–related items for RCTs. General reporting items from the original CONSORT 2010 and SPIRIT 2013 checklists were not assessed.

### Comparison With Previous Work

We are aware of only one published systematic scoping review on the application of AI in community-based primary health care [3]. The authors highlighted a gap in the development and implementation of AI in primary care. However, reporting quality was not assessed previous study [3]. This systematic review is the first focusing on primary care and indicating the need for more transparent design, conduct, and analysis of AI interventions in primary care.

### Implications for Research and Practice

To apply AI interventions in primary care, accurate and sufficient information is essential to guide the standardized performance of clinical treatment. The findings of this study have several implications for clinical research and practice, concerning AI safety, reliability, and reproducibility, as outlined below.

First, reporting on AI performance errors needs to be improved. As software, AI systems are likely to undergo multiple iterations and updates [10]. For example, although LLMs have successfully answered medical licensing exam questions [63], they still produce errors when stating facts or synthesizing data from medical literature [64]. Since LLMs still have many flaws, performance error reporting is essential for continuous updating. The lack of performance error reporting may lead to misdiagnosis or incorrect treatment recommendations, ultimately jeopardizing patient safety [65]. It is crucial to specify the types of performance errors, the process of identifying them, and how updated versions correct these errors during the trial.

Second, the reporting on the section of input-data setting deserves special attention. AI interventions applied to primary care should provide comprehensive information for input-data settings, including parameter selection and preprocessing before analysis by the AI system. Moreover, during the input-data setting, the inclusion and exclusion criteria should be clearly defined, along with an adequate investigation of patient characteristics relevant to the disease type. It is necessary to report these items completely to ensure the replicability of the intervention beyond the trials in real-world circumstances [19]. It also supports investigators in identifying whether input-data-handling procedures were standardized across trial sites [17]. Overall, the AI system should establish an optimal input set that includes a wide range of parameters and attribute combinations [66]. For example, ML prediction models can only accurately predict the specific outcomes after knee arthroplasty while the ability to predict more complex outcomes remains inaccuracy [67]. Thus, ML requires comprehensive patient-related indicators and the identification of specific patterns that are suitable for ML analysis. This underscores the importance of high-quality input data setting as a fundamental prerequisite for optimizing ML capabilities in clinical applications. Finally, trials should report the prespecified conditions, particularly if minimum requirements for input data are not met. Failing to transparently report how input data were acquired and selected can compromise the representativeness and generalizability of the AI model, potentially leading to critical errors in AI-driven decision-making, which may adversely affect patient safety and clinical outcomes.[17]

In particular, reporting on assessment and handling of input data needs to be improved. The reporting of AI intervention should mention the amount of poor-quality input data, as well as how this was identified and handled [17]. Poor-quality or unavailable input data can negatively impact the success of AI algorithms and the AI’s performance and effectiveness. It is essential to have a plan in place for handling such scenarios, such as implementing data cleaning techniques, using data imputation methods, or seeking alternative data sources.[10] A study on cardiovascular imaging pointed out that the quality and preprocessing of input data such as echocardiography, cardiac magnetic resonance imaging, and cardiac computed tomography should be evaluated before a model can be trained using deep learning techniques [68]. Otherwise, the black-box nature of AI models may lead to a lack of trust among clinicians and sponsors [69,70].

Finally, reporting regarding access to and reuse of AI intervention and its code needs to be specified. Human-AI interaction promotes the capabilities of AI systems, as it leverages human expertise to ensure the practical application of AI models. A review on ML highlights three reasons for the necessity of human involvement: real-world problems are inherently complex, ML methods often lack explainability, and AI outputs may not always align with clinical expectations or disease judgment [71]. It is imperative that health care professionals can validate and refine AI methodologies to ensure they are clinically applicable [72,73]. Therefore, specifying whether and how the AI intervention and its underlying code can be accessed by designated health care professionals is crucial. Clear reporting on the accessibility and transparency of AI systems further helps clinicians understand their functionality, enhancing trust in their outputs and fostering the development of user-friendly interfaces, and ensuring the safe and effective use of AI in clinical practice.

### Conclusion

Our study focused on AI intervention in primary care, providing a first systematic review of qualified reporting of RCTs using AI interventions. This study indicated significant gaps between the reporting guidelines and published articles and underscored the crucial items and aspects that were frequently overlooked in the reporting framework. It is said that the whole of medicine depends on the transparent reporting of clinical trials [74]. Our findings may contribute to the enhancement of quality standards in AI research in primary care trials and help future clinical AI investigators to design, conduct, and analysis of higher-quality AI interventions for primary care.

## Data Availability

The datasets generated or analyzed during this study are available from the corresponding author on reasonable request.

## Appendix

Multimedia Appendix 1 PRISMA (Preferred Reporting Items for Systematic Reviews and Meta-Analysis) checklist.

Multimedia Appendix 2 Search strategies.

Multimedia Appendix 3 List and characteristics of included randomized controlled trials and protocols.

Multimedia Appendix 4 Association between primary outcome result status and the reporting quality of each item.

Multimedia Appendix 5 Association between trial characteristics and the percentage of adequately reported items.

## Abbreviations

AI: artificial intelligence
CDSS: clinical decision support system
CONSORT-AI: Consolidated Standards of Reporting Trials–Artificial Intelligence
LLM: large language model
ML: machine learning
PRISMA: Preferred Reporting Items for Systematic Reviews and Meta-Analysis
RCT: randomized controlled trial
SPIRIT-AI: Standard Protocol Items: Recommendations for Interventional Trials–Artificial Intelligence
