# Supplementary material for: Reporting Quality of AI Intervention in Randomized Controlled Trials in Primary Care: Systematic Review and Meta-Epidemiological Study
Source: J Med Internet Res. 2025 Feb 25;27:e56774. doi: 10.2196/56774 (PMC11897677; doi:10.2196/56774)
Supplement: Multimedia Appendix 2 [file jmir_v27i1e56774_app2.docx]

### Part 1 Identification of studies via databases and registers (up to 30th Nov 2024)

### PubMed

| #1 | ((((((((((((primary care) OR (primary health care)) OR (general practice)) OR (family medicine)) OR (family doctor)) OR (family practice)) OR (public health)) OR (home nurse)) OR (home care services)) OR (community health services)) OR (rural health services)) OR (general practitioners)) OR (community medicine) | 11,327,258 |
| --- | --- | --- |
| #2 | (((((((((((((((((AI[Title/Abstract]) OR (artificial intelligence[Title/Abstract])) OR (deep learning[Title/Abstract])) OR (machine learning[Title/Abstract])) OR (SVM[Title/Abstract])) OR (neural network[Title/Abstract])) OR (decision tree[Title/Abstract])) OR (random forest[Title/Abstract])) OR (support vector machine[Title/Abstract])) OR (supervised learning[Title/Abstract])) OR (computer learning[Title/Abstract])) OR (computer intelligence[Title/Abstract])) OR (natural language processing[Title/Abstract])) OR (artificial learning[Title/Abstract])) OR (machine intelligence[Title/Abstract])) OR (gradient boosting machine[Title/Abstract])) OR (decision support[Title/Abstract])) OR (expert system[Title/Abstract]) | 384,192 |
| #3 | (((((((((randomized[Title/Abstract]) OR (randomised[Title/Abstract])) OR (RCT[Title/Abstract])) OR (randomized controlled trials[Title/Abstract])) OR (random allocation[Title/Abstract])) OR (randomly allocated[Title/Abstract])) OR (cross over[Title/Abstract])) OR (placebo[Title/Abstract])) OR (single blind*[Title/Abstract])) OR (double blind*[Title/Abstract]) | 1,035,137 |
| #4 | #1 and #2 and #3 | 5,660 |

### Embase

| #1 | 'artificial intelligence'/exp | 121,858 |
| --- | --- | --- |
| #2 | 'machine learning'/exp | 534,095 |
| #3 | 'machine learning':ab,ti OR 'artificial intelligence':ab,ti OR 'random forest':ab,ti OR 'decision tree':ab,ti OR 'support vector machine':ab,ti OR 'gradient boosting machine':ab,ti OR 'expert system':ab,ti OR 'ai':ab,ti OR 'computer intelligence':ab,ti OR 'natural language processing':ab,ti OR 'computer vision system':ab,ti OR 'deep learning':ab,ti OR 'neural network':ab,ti | 405,492 |
| #4 | #1 OR #2 OR #3 | 710,483 |
| #5 | 'randomized controlled trial':ab,ti OR rct:ab,ti OR randomized:ab,ti OR randomization:ab,ti OR 'randomly allocated':ab,ti OR 'crossover procedure':ab,ti OR placebo:ab,ti OR ai:ab,ti OR 'single blind*':ab,ti OR 'double blind*':ab,ti | 1,455,777 |
| #6 | 'randomized controlled trial'/exp | 858,458 |
| #7 | #5 OR #6 | 1,692,960 |
| #8 | 'primary medical care'/exp OR 'primary health care'/exp OR 'general practice'/exp OR 'family medicine'/exp | 312,751 |
| #9 | 'primary medical care':ab,ti OR 'primary health care':ab,ti OR 'general practice':ab,ti OR 'family medicine':ab,ti OR 'rural health  care':ab,ti OR 'public health':ab,ti OR 'home nurse':ab,ti OR 'community health service':ab,ti OR 'community medicine':ab,ti OR 'home care services':ab,ti OR 'general practitioner':ab,ti | 563,608 |
| #10 | #8 OR #9 | 791,040 |
| #11 | #4 AND #7 AND #10 | 2,197 |

### Cochrane

| #1 | ("primary-care"):ti,ab,kw OR ("primary health care"):ti,ab,kw OR ("community health nurse"):ti,ab,kw OR ("general practice"):ti,ab,kw OR ("family medicine"):ti,ab,kw | 31745 |
| --- | --- | --- |
| #2 | MeSH descriptor: [Primary Health Care] explode all trees | 12224 |
| #3 | MeSH descriptor: [General Practice] explode all trees | 3165 |
| #4 | ("artificial intelligence"):ti,ab,kw OR ("machine learning"):ti,ab,kw OR ("neural network theory"):ti,ab,kw OR (decision tree):ti,ab,kw OR ("natural language processing"):ti,ab,kw | 6220 |
| #5 | MeSH descriptor: [Artificial Intelligence] explode all trees | 3435 |
| #6 | MeSH descriptor: [Machine Learning] explode all trees | 1082 |
| #7 | ("randomized controlled clinical trial"):ti,ab,kw OR (RCT):ti,ab,kw OR ("single blind method"):ti,ab,kw OR ("double blind method"):ti,ab,kw OR ("randomization"):ti,ab,kw | 1436448 |
| #8 | MeSH descriptor: [Randomized Controlled Trial as Topic] explode all trees | 58312 |
| #9 | #1 or #2 or #3 | 37460 |
| #10 | #4 or #5 or #6 | 8118 |
| #11 | #7 or #8 | 1436544 |
| #12 | #9 and #10 and #11 | 188 |

### Part 2 Identification of studies via other databases (up to 30th Nov 2024).

### Medline

| 1 | Artificial Intelligence/ or DATA MINING/ or EXPERT SYSTEMS/ or fuzzy logic/ or exp Machine Learning/ or NATURAL LANGUAGE PROCESSING/ or Support Vector Machine/ or "neural networks (computer)"/ | 184356 |
| --- | --- | --- |
| 2 | ("artificial intelligence*" or "computational intelligence*" or "machine intelligence*" or "automated reasoning" or "bayesian network*" or "bayes network*" or "naive bayes" or "bayesian learning" or "computer heuristic*" or "computer reasoning" or "data mining" or "text mining" or "expert system*" or "fuzzy logic" or "fuzzy cognitive" or "knowledge representation*" or "knowledge acquisition*" or "machine learning" or "learning machine*" or "natural language processing*" or "neural network*" or "deep learning" or "support vector*" or "hidden markov model*" or "random forest*" or "random decision forest*" or "supervised learning" or "unsupervised learning" or "autoencoder*" or "Generative adversarial network*" or "reservoir computing" or "shallow learning" or "echo state network*" or "case-based reasoning" or "metaheuristic*" or "soft computing" or "approximate reasoning" or "evolutionary computing" or "genetic algorithm*" or "bio-inspired algorithm*").ti.  or ("artificial intelligence*" or "computational intelligence*" or "machine intelligence*" or "automated reasoning" or "bayesian network*" or "bayes network*" or "naive bayes" or "bayesian learning" or "computer heuristic*" or "computer reasoning" or "data mining" or "text mining" or "expert system*" or "fuzzy logic" or "fuzzy cognitive" or "knowledge representation*" or "knowledge acquisition*" or "machine learning" or "learning machine*" or "natural language processing*" or "neural network*" or "deep learning" or "support vector*" or "hidden markov model*" or "random forest*" or "random decision forest*" or "supervised learning" or "unsupervised learning" or "autoencoder*" or "Generative adversarial network*" or "reservoir computing" or "shallow learning" or "echo state network*" or "case-based reasoning" or "metaheuristic*" or "soft computing" or "approximate reasoning" or "evolutionary computing" or "genetic algorithm*" or "bio-inspired algorithm*").ab.  or ("artificial intelligence*" or "computational intelligence*" or "machine intelligence*" or "automated reasoning" or "bayesian network*" or "bayes network*" or "naive bayes" or "bayesian learning" or "computer heuristic*" or "computer reasoning" or "data mining" or "text mining" or "expert system*" or "fuzzy logic" or "fuzzy cognitive" or "knowledge representation*" or "knowledge acquisition*" or "machine learning" or "learning machine*" or "natural language processing*" or "neural network*" or "deep learning" or "support vector*" or "hidden markov model*" or "random forest*" or "random decision forest*" or "supervised learning" or "unsupervised learning" or "autoencoder*" or "Generative adversarial network*" or "reservoir computing" or "shallow learning" or "echo state network*" or "case-based reasoning" or "metaheuristic*" or "soft computing" or "approximate reasoning" or "evolutionary computing" or "genetic algorithm*" or "bio-inspired algorithm*").kw. | 376422 |
| 3 | \| 1 or 2 \| \| --- \| | 413479 |
| 4 | Primary Health Care/ or exp General Practice/ or Physicians, Family/ or General Practitioners/ or Physicians, Primary Care/ or exp Group Practice/ or Ambulatory Care/ or exp Community Health Services/ or exp Ambulatory Care Facilities/ or exp Rural Health Services/ or exp nurse practitioners/ or Community Health Workers/ or Community Medicine/ or home nursing/ or primary care nursing/ or nurses, community health/ or nurses, public health/ or nurse clinicians/ or nurse midwives/ or Pharmacists/ or exp Home Care Services/ or Hospices/ or exp Community Health Nursing/ or Office Visits/ or House Calls/ or emergency medical technicians/ or exp Emergency Service, Hospital/ or pharmacy technicians/ or emergency medical services/ or emergency services, psychiatric/ or Family Health/ | 848696 |
| 5 | ((primary adj3 care) or ("primary healthcare" or "primary health" or "first line" or "family healthcare") or ((family or general* or group) adj2 (doctor* or physician* or pract* or medicine or nurs*)) or (rural adj3 (physician* or practice or service* or hospital*)) or generalist* or (ambulatory adj2 (care or clinic* or service*)) or (health adj3 (center* or centre*)) or consult* or (visit* adj3 (clinic* or care or outpatient* or office*)) or (community adj3 (care or worker* or service* or nurs* or pract*)) or (social adj2 (worker* or service*)) or practitioner* or "clinical practice*" or pharmacist* or dietitian* or hospice* or ((home or domicil*) adj3 (care or healthcare or nurs* or rehabilit* or service* or visiting or visit?)) or homecare* or (nurse adj1 (rehabilitator* or clinician*)) or paramedic* or ((emergency or emergencies or trauma) adj3 (department* or service* or outpatient* or ward* or room* or unit or units or center* or centre* or physician* or nurse*)) or "ED" or "family health" or midwi*).ti. or ((primary adj3 care) or ("primary healthcare" or "primary health" or "first line" or "family healthcare") or ((family or general* or group) adj2 (doctor* or physician* or pract* or medicine or nurs*)) or (rural adj3 (physician* or practice or service* or hospital*)) or generalist* or (ambulatory adj2 (care or clinic* or service*)) or (health adj3 (center* or centre*)) or consult* or (visit* adj3 (clinic* or care or outpatient* or office*)) or (community adj3 (care or worker* or service* or nurs* or pract*)) or (social adj2 (worker* or service*)) or practitioner* or "clinical practice*" or pharmacist* or dietitian* or hospice* or ((home or domicil*) adj3 (care or healthcare or nurs* or rehabilit* or service* or visiting or visit?)) or homecare* or (nurse adj1 (rehabilitator* or clinician*)) or paramedic* or ((emergency or emergencies or trauma) adj3 (department* or service* or outpatient* or ward* or room* or unit or units or center* or centre* or physician* or nurse*)) or "ED" or "family health" or midwi*).ab. or ((primary adj3 care) or ("primary healthcare" or "primary health" or "first line" or "family healthcare") or ((family or general* or group) adj2 (doctor* or physician* or pract* or medicine or nurs*)) or (rural adj3 (physician* or practice or service* or hospital*)) or generalist* or (ambulatory adj2 (care or clinic* or service*)) or (health adj3 (center* or centre*)) or consult* or (visit* adj3 (clinic* or care or outpatient* or office*)) or (community adj3 (care or worker* or service* or nurs* or pract*)) or (social adj2 (worker* or service*)) or practitioner* or "clinical practice*" or pharmacist* or dietitian* or hospice* or ((home or domicil*) adj3 (care or healthcare or nurs* or rehabilit* or service* or visiting or visit?)) or homecare* or (nurse adj1 (rehabilitator* or clinician*)) or paramedic* or ((emergency or emergencies or trauma) adj3 (department* or service* or outpatient* or ward* or room* or unit or units or center* or centre* or physician* or nurse*)) or "ED" or "family health" or midwi*).kw. | 1556271 |
| 6 | 4 or 5 | 2008100 |
| 7 | exp Randomized Controlled Trial/ or Controlled Clinical Trial/ or Pragmatic Clinical Trial/ or Equivalence Trial/ | 719125 |
| 8 | ("randomized controlled trial*" or "randomized trial*" or "RCT*" or "randomized study*" or "randomized clinical trial*" or "random allocation*" or "randomized*" or "clinical trial*" or "clinic* trial" or "random* NEAR(3) trial" or "random* clinical trial" or "RCT" or "randomized study").ti. or ("randomized controlled trial*" or "randomized trial*" or "RCT*" or "randomized study*" or "randomized clinical trial*" or "random allocation*" or "andomized*" or "clinical trial*" or "clinic* trial" or "random* NEAR(3) trial" or "random* clinical trial" or "RCT" or "randomized study").ab. or ("randomized controlled trial*" or "randomized trial*" or "RCT*" or "randomized study*" or "randomized clinical trial*" or "ranrdom allocation*" or "randomized*" or "clinical trial*" or "clinic* trial" or "random* NEAR(3) trial" or "random* clinical trial" or "RCT" or "randomized study").kw. | 1145441 |
| 9 | 7 or 8 | 1483396 |
| 10 | 3 and 6 and 9 | 1438 |

### Web of science

| 1 | TS=("artificial intelligence*" or "computational intelligence*" or "machine intelligence*" or "automated reasoning" or "bayesian network*" or "bayes network*" or "naive bayes" or "bayesian learning" or "computer heuristic*" or "computer reasoning" or "data mining" or "text mining" or "expert system*" or "fuzzy logic" or "fuzzy cognitive" or "knowledge representation*" or "knowledge acquisition*" or "machine learning" or "learning machine*" or "natural language processing*" or "neural network*" or "deep learning" or "support vector*" or "hidden markov model*" or "random forest*" or "random decision forest*" or "supervised learning" or "unsupervised learning" or "autoencoder*" or "Generative adversarial network*" or "reservoir computing" or "shallow learning" or "echo state network*" or "case-based reasoning" or "metaheuristic*" or "soft computing" or "approximate reasoning" or "evolutionary computing" or "genetic algorithm*" or "bio-inspired algorithm*") | [1,924,646](https://www.webofscience.com/wos/woscc/summary/ee5ac2f3-335f-4a09-9439-8222d5adc301-012fa8c7c9/relevance/1) |
| --- | --- | --- |
| 2 | TS=((primary NEAR/2 care) or ("primary healthcare" or "primary health" or "first line" or "family healthcare") or ((family or general* or group) NEAR/1 (doctor* or physician* or pract* or medicine or nurs*)) or (rural NEAR/2 (physician* or practice or service* or hospital*)) or generalist* or (ambulatory NEAR/1 (care or clinic* or service*)) or (health NEAR/2 (center* or centre*)) or consult* or (visit* NEAR/2 (clinic* or care or outpatient* or office*)) or (community NEAR/2 (care or worker* or service* or nurs* or pract*)) or (social NEAR/1 (worker* or service*)) or practitioner* or "clinical practice*" or pharmacist* or dietitian* or hospice* or ((home or domicil*) NEAR/2 (care or healthcare or nurs* or rehabilit* or service* or visiting or visit?)) or homecare* or (nurse NEAR/0 (rehabilitator* or clinician*)) or paramedic* or ((emergency or emergencies or trauma) NEAR/2 (department* or service* or outpatient* or ward* or room* or unit or units or center* or centre* or physician* or nurse*)) or "ED" or "family health" or midwi*:ti,ab,kw) | [1,829,811](https://www.webofscience.com/wos/woscc/summary/1c4f3385-240f-4ae8-9526-0a3c07f5ce2e-012fa8f6f6/relevance/1) |
| 3 | TS=("randomized controlled trial*" or "randomized trial*" or "RCT*" or "randomized study*" or "randomized clinical trial*" or "random allocation*" or "randomized*" or "clinical trial*" or "clinic* trial" or "random* NEAR(3) trial" or "random* clinical trial" or "RCT" or "randomized study") | [1,443,606](https://www.webofscience.com/wos/woscc/summary/c1921146-f037-4c44-9e03-4a7d591bb829-012fa9ac61/relevance/1) |
| 4 | 1 and 2 and 3 | [1,627](https://www.webofscience.com/wos/woscc/summary/23013db3-a956-40f8-9639-1548e2ee982b-012fa9d83c/relevance/1) |

### CINAHL

| S1 | MH Artificial Intelligence+ OR MH Data Mining | 39,649 |
| --- | --- | --- |
| S2 | TI ( "artificial intelligence*" or "computational intelligence*" or "machine intelligence*" or "automated reasoning" or "bayesian network*" or "bayes network*" or "naive bayes" or "bayesian learning" or "computer heuristic*" or "computer reasoning" or "data mining" or "text mining" or "expert system*" or "fuzzy logic" or "fuzzy cognitive" or "knowledge representation*" or "knowledge acquisition*" or "machine learning" or "learning machine*" or "natural language processing*" or "neural network*" or "deep learning" or "support vector*" or "hidden markov model*" or "random forest*" or "random decision forest*" or "supervised learning" or "unsupervised learning" or "autoencoder*" or "Generative adversarial network*" or "reservoir computing" or "shallow learning" or "echo state network*" or "case-based reasoning" or "metaheuristic*" or "soft computing" or "approximate reasoning" or "evolutionary computing" or "genetic algorithm*" or "bio-inspired algorithm*" ) OR AB ( "artificial intelligence*" or "computational intelligence*" or "machine intelligence*" or "automated reasoning" or "bayesian network*" or "bayes network*" or "naive bayes" or "bayesian learning" or "computer heuristic*" or "computer reasoning" or "data mining" or "text mining" or "expert system*" or "fuzzy logic" or "fuzzy cognitive" or "knowledge representation*" or "knowledge acquisition*" or "machine learning" or "learning machine*" or "natural language processing*" or "neural network*" or "deep learning" or "support vector*" or "hidden markov model*" or "random forest*" or "random decision forest*" or "supervised learning" or "unsupervised learning" or "autoencoder*" or "Generative adversarial network*" or "reservoir computing" or "shallow learning" or "echo state network*" or "case-based reasoning" or "metaheuristic*" or "soft computing" or "approximate reasoning" or "evolutionary computing" or "genetic algorithm*" or "bio-inspired algorithm*" ) OR MW ( "artificial intelligence*" or "computational intelligence*" or "machine intelligence*" or "automated reasoning" or "bayesian network*" or "bayes network*" or "naive bayes" or "bayesian learning" or "computer heuristic*" or "computer reasoning" or "data mining" or "text mining" or "expert system*" or "fuzzy logic" or "fuzzy cognitive" or "knowledge representation*" or "knowledge acquisition*" or "machine learning" or "learning machine*" or "natural language processing*" or "neural network*" or "deep learning" or "support vector*" or "hidden markov model*" or "random forest*" or "random decision forest*" or "supervised learning" or "unsupervised learning" or "autoencoder*" or "Generative adversarial network*" or "reservoir computing" or "shallow learning" or "echo state network*" or "case-based reasoning" or "metaheuristic*" or "soft computing" or "approximate reasoning" or "evolutionary computing" or "genetic algorithm*" or "bio-inspired algorithm*" ) | 57,688 |
| S3 | S1 OR S2 | 67,401 |
| S4 | (MH "Primary Health Care") OR (MH "Family Practice") OR (MH "Physicians, Family") OR (MH "Group Practice+") OR (MH "Ambulatory Care Facilities") OR (MH "Ambulatory Care Facilities+") OR (MH "Community Health Centers+") OR (MH "Community Health Services+") OR (MH "Community Health Workers") OR (MH "Rural Health Personnel") OR (MH "Rural Health Nursing") OR (MH "Rural Health Centers") OR (MH "Rural Health Services") OR (MH "Nurse Practitioners+") OR (MH "Community Medicine") OR (MH "Community Health Nursing+") OR (MH "Clinical Nurse Specialists") OR (MH "Nurse Midwives") OR (MH "Nurse Practitioners") OR (MH "Pharmacists") OR (MH "Home Health Care+") OR (MH "Home Nursing") OR (MH "Hospices") OR (MH "Office Visits") OR (MH "Home Visits") OR (MH "Emergency Service+") OR (MH "Physicians, Emergency") OR (MH "Pharmacy Technicians") OR (MH "Emergency Medical Services+") OR (MH "Family Health") | 860,224 |
| S5 | TI ( ( (primary N2 care) or ("primary healthcare" or "primary health" or "first line" or "family healthcare") or ((family or general* or group) N1 (doctor* or physician* or pract* or medicine or nurs*)) or (rural N2 (physician* or practice or service* or hospital*)) or generalist* or (ambulatory N1 (care or clinic* or service*)) or (health N2 (center* or centre*)) or consult* or (visit* N2 (clinic* or care or outpatient* or office*)) or (community N2 (care or worker* or service* or nurs* or pract*)) or (social N1 (worker* or service*)) or practitioner* or "clinical practice*" or pharmacist* or dietitian* or hospice* or ((home or domicil*) N2 (care or healthcare or nurs* or rehabilit* or service* or visiting or visit?))or homecare* or (nurse N0 (rehabilitator* or clinician*)) or paramedic* or ((emergency or emergencies or trauma) N2 (department* or service* or outpatient* or ward* or room* or unit or units or center* or centre* or physician* or nurse*)) or "ED" or "family health" or midwi* ) ) OR AB ( ( (primary N2 care) or ("primary healthcare" or "primary health" or "first line" or "family healthcare") or ((family or general* or group) N1 (doctor* or physician* or pract* or medicine or nurs*)) or (rural N2 (physician* or practice or service* or hospital*)) or generalist* or (ambulatory N1 (care or clinic* or service*)) or (health N2 (center* or centre*)) or consult* or (visit* N2 (clinic* or care or outpatient* or office*)) or (community N2 (care or worker* or service* or nurs* or pract*)) or (social N1 (worker* or service*)) or practitioner* or "clinical practice*" or pharmacist* or dietitian* or hospice* or ((home or domicil*) N2 (care or healthcare or nurs* or rehabilit* or service* or visiting or visit?))or homecare* or (nurse N0 (rehabilitator* or clinician*)) or paramedic* or ((emergency or emergencies or trauma) N2 (department* or service* or outpatient* or ward* or room* or unit or units or center* or centre* or physician* or nurse*)) or "ED" or "family health" or midwi* ) ) OR SU ( ( (primary N2 care) or ("primary healthcare" or "primary health" or "first line" or "family healthcare") or ((family or general* or group) N1 (doctor* or physician* or pract* or medicine or nurs*)) or (rural N2 (physician* or practice or service* or hospital*)) or generalist* or (ambulatory N1 (care or clinic* or service*)) or (health N2 (center* or centre*)) or consult* or (visit* N2 (clinic* or care or outpatient* or office*)) or (community N2 (care or worker* or service* or nurs* or pract*)) or (social N1 (worker* or service*)) or practitioner* or "clinical practice*" or pharmacist* or dietitian* or hospice* or ((home or domicil*) N2 (care or healthcare or nurs* or rehabilit* or service* or visiting or visit?))or homecare* or (nurse N0 (rehabilitator* or clinician*)) or paramedic* or ((emergency or emergencies or trauma) N2 (department* or service* or outpatient* or ward* or room* or unit or units or center* or centre* or physician* or nurse*)) or "ED" or "family health" or midwi* ) ) | 1,026,716 |
| S6 | S4 OR S5 | 1,420,559 |
| S7 | MH Randomized Controlled Trial OR MH Controlled Clinical Trial/ OR MH Pragmatic Clinical Trial OR Equivalence Trial | 519 |
| S8 | TI ( ("randomized controlled trial*" or "randomized trial*" or "RCT*" or "randomized study*" or "randomized clinical trial*" or "random allocation*" or "randomized*" or "clinical trial*" or "clinic* trial" or "random* NEAR(3) trial" or "random* clinical trial" or "RCT" or "randomized study") ) OR AB ( ("randomized controlled trial*" or "randomized trial*" or "RCT*" or "randomized study*" or "randomized clinical trial*" or "random allocation*" or "randomized*" or "clinical trial*" or "clinic* trial" or "random* NEAR(3) trial" or "random* clinical trial" or "RCT" or "randomized study") ) OR SU ( ("randomized controlled trial*" or "randomized trial*" or "RCT*" or "randomized study*" or "randomized clinical trial*" or "random allocation*" or "randomized*" or "clinical trial*" or "clinic* trial" or "random* NEAR(3) trial" or "random* clinical trial" or "RCT" or "randomized study") ) | 520,578 |
| S9 | S7 OR S8 | 520,669 |
| S10 | S5 AND S6 AND S9 | 601 |

REFERENCE

1. Abbasgholizadeh Rahimi S, Légaré F, Sharma G, Archambault P, Zomahoun HTV, Chandavong S, et al. Application of Artificial Intelligence in Community-Based Primary Health Care: Systematic Scoping Review and Critical Appraisal. J Med Internet Res. 2021 Sep 3;23(9):e29839. doi: 10.2196/29839. PMID: 34477556.
