# Supplementary material for: Reporting Quality of AI Intervention in Randomized Controlled Trials in Primary Care: Systematic Review and Meta-Epidemiological Study
Source: J Med Internet Res. 2025 Feb 25;27:e56774. doi: 10.2196/56774 (PMC11897677; doi:10.2196/56774)
Supplement: Multimedia Appendix 3 [file jmir_v27i1e56774_app3.docx]

**List and characteristics of included randomized controlled trials and protocols**

| **No.** | **Type** | **Study name** | **Trial identifier** | **First author** | **Title** | **Journal** | **Publication year** | **Healthcare issue** | **Primary outcome** | **Country** | **Proportion of adequately reported items** | **Article DOI Link** |
| --- | --- | --- | --- | --- | --- | --- | --- | --- | --- | --- | --- | --- |
| 1 | RCT | iSmartHyp | NCT03288142^a^ | Persell SD[23] | Effect of home blood pressure monitoring via a smartphone hypertension coaching application or tracking application on adults with uncontrolled hypertension a randomized clinical trial | JAMA Netw Open | 2020 | Hypertension | Systolic blood pressure | USA | 9/14 | <https://doi.org/10.1001/jamanetworkopen.2020.0255> |
| 2 | RCT | AMP | NCT02865967 | Seol HY[24] | Artificial intelligence-assisted clinical decision support for childhood asthma management: a randomized clinical trial | PLoS One | 2021 | Asthma | Occurrence of asthma exacerbation | USA | 9/14 | <https://doi.org/10.1371/journal.pone.0255261> |
| 3 | RCT | EAGLE | NCT04000087^b^ | Yao X[25] | Artificial intelligence–enabled electrocardiograms for identification of patients with low ejection fraction: a pragmatic, randomized clinical trial | Nat Med | 2021 | Low ejection fraction | The diagnosis of low EF | USA | 13/14 | <https://doi.org/10.1038/s41591-021-01335-4> |
| 4 | RCT | Not available | UMIN000041037 | Itoh N[26] | Evaluation of the effect of patient education and strengthening exercise therapy using a mobile messaging app on work productivity in japanese patients with chronic low back pain: open-label, randomized, parallel-group trial | JMIR Mhealth Uhealth | 2022 | Low back pain | The change in work productivity at week 12 | Japan | 8/14 | <https://doi.org/10.2196/35867> |
| 5 | RCT | REACT (AI CBT) | NCT02464449 | Piette JD[27] | Patient-centered pain care using artificial intelligence and mobile health tools: a randomized comparative effectiveness trial | JAMA Intern Med | 2022 | Pain care | The Roland Morris Disability Questionnaire (RMDQ; range 0-24) | USA | 10/14 | <https://doi.org/10.1001/jamainternmed.2022.3178> |
| 6 | RCT | EAGLE | NCT04000087 | Rushlow DR[28] | Clinician adoption of an artificial intelligence algorithm to detect left ventricular systolic dysfunction in primary care. | Mayo Clin Proc | 2022 | Left ventricular systolic dysfunction | The impact of adoption on the clinical outcome of low EF, and the provider characteristics associated with adoption | USA | 10/14 | <https://doi.org/10.1016/j.mayocp.2022.04.008> |
| 7 | RCT | Not available | NCT03984773 | Manz CR[29] | Long-term effect of machine learning-triggered behavioral nudges on serious illness conversations and end-of-life outcomes among patients with cancer: a randomized clinical trial | JAMA Oncol | 2023 | Cancer care delivery | Serious Illness Conversations (SIC) rates for all and high-risk patient encounters | USA | 7/14 | <https://doi.org/10.1001/jamaoncol.2022.6303> |
| 8 | RCT | ColonCADe | NCT04555135 | Wei MT[30] | Evaluation of computer-aided detection during colonoscopy in the community (ai-see): a multicenter randomized clinical trial | Am J Gastroenterol | 2023 | Polyp detection | Adenomas per colonoscopy and adenomas per extraction | USA | 7/14 | <https://doi.org/10.14309/ajg.0000000000002239> |
| 9 | RCT | Not available | ISRCTN13817256^c^ | Yang J, [31] | Effects of a feedback intervention on antibiotic prescription control in primary care institutions based on a health information system: a cluster randomized cross-over controlled trial | J Glob Antimicrob Resist | 2023 | Antibiotic prescription | The 10-day inappropriate antibiotic prescription rate | China | 9/14 | <https://doi.org/10.1016/j.jgar.2023.02.006> |
| 10 | RCT | Not available | NCT05118035 | Lin C-S[32] | AI-enabled electrocardiography alert intervention and all-cause mortality: a pragmatic randomized clinical trial | Nat Med | 2024 | Cardiovascular disease | All-cause mortality | China | 14/14 | https://doi.org/10.1038/s41591-024-02961-4 |
| 11 | RCT | AID-ME | NCT04655924 | Benrimoh D[33] | Artificial Intelligence in Depression - Medication Enhancement (AID-ME): a cluster randomized trial of a deep learning enabled clinical decision support system for personalized depression treatment selection and management | medRxiv | 2024 | Depression | Remission of depressive symptoms | Canada | 10/14 | <https://doi.org/10.21203/rs.3.rs-4587945/v1> |
| 12 | RCT | [Dej@lo](mailto:Dej@lo) | NCT03445507^d^ | Olano-Espinosa E[34] | Effectiveness of a conversational chatbot (Dejal@bot) for the adult population to quit smoking: pragmatic, multicenter, controlled, randomized clinical trial in primary care | JMIR Mhealth Uhealth | 2022 | Quit Smoking | Continuous abstinence at 6 months | Spain | 9/14 | <https://doi.org/10.2196/34273> |
| 13 | RCT | selfBACK | NCT03798288^e^ | Sandal LF[35] | Effectiveness of app-delivered, tailored self-management support for adults with lower back pain-related disability: a selfback randomized clinical trial | JAMA Intern Med | 2021 | Low back pain | The mean difference in RMDQ scores between the intervention group and control group at 3 months. | Denmark | 7/14 | <https://doi.org/10.1001/jamainternmed.2021.4097> |
| 14 | RCT | Not available | NCT06157944 | Goh E[36] | Large language model influence on diagnostic reasoning: a randomized clinical trial | JAMA Netw Open | 2024 | Diagnostic reasoning | The final score as a percentage across all components of the structured reflection tool | USA | 7/14 | <https://doi.org/10.1001/jamanetworkopen.2024.40969> |
| 15 | RCT | SupportPrim | ISRCTN17927832 | Granviken F [37] | Personalised decision support in the management of patients with musculoskeletal pain in primary physiotherapy care: a cluster randomised controlled trial (the SupportPrim project) | Pain | 2024 | Musculoskeletal pain | Self-reported GPE (Global Perceived Effect) and clinically important improvement in function measured by the Patient-Specific Functional Scale (PSFS) | Norway | 9/14 | <https://doi.org/10.1097/j.pain.0000000000003456> |
| 16 | RCT | CAD | NCT05963724 | Thiruvengadam NR [38] | The efficacy of real-time computer-aided detection of colonic neoplasia in community practice: a pragmatic randomized controlled trial | Clin Gastroenterol Hepatol | 2024 | Colonic Neoplasia | Adenoma detection rate (ADR) | USA | 7/14 | <https://doi.org/10.1016/j.cgh.2024.02.021> |
| 17 | RCT | MIVA | NCT05081011 | Nayak A [39] | Use of voice-based conversational artificial intelligence for basal insulin prescription management among patients with type 2 diabetes: a randomized clinical trial | JAMA Netw Open | 2023 | Type 2 Diabetes | Time to optimal insulin dose | USA | 9/14 | <https://doi.org/10.1001/jamanetworkopen.2023.40232> |
| 18 | RCT | SOY+ | NCT04802018 | Alcoceba-Herrero I [40] | Randomized controlled trial to assess the feasibility of a novel clinical decision support system based on the automatic generation of alerts through remote patient monitoring | J Clin Med | 2024 | COVID-19 | Progression to pneumonia, need for intensive care unit (ICU) admission, need for invasive mechanical ventilation, and mortality rate | Spain | 9/14 | <https://doi.org/10.3390/jcm13195974> |
| 19 | RCT | Timely | NCT04909671 | Ortiz O [41] | An artificial intelligence-assisted system versus white light endoscopy alone for adenoma detection in individuals with Lynch syndrome (TIMELY): an international, multicentre, randomised controlled trial | Lancet Gastroenterol Hepatol | 2024 | Adenoma | The mean number of adenomas per colonoscopy | Spain | 8/14 | <https://doi.org/10.1016/S2468-1253(24)00187-0> |
| 20 | Protocol | iSmartHyp | NCT03288142^a^ | Persell SD [42] | Design of a randomized controlled trial comparing a mobile phone-based hypertension health coaching application to home blood pressure monitoring alone: the smart hypertension control study | Contemp Clin Trials | 2018 | Hypertension | Systolic blood pressure at six months | USA | 10/15 | <https://doi.org/10.1016/j.cct.2018.08.013> |
| 21 | Protocol | Not available | DERR1-10.2196/13151 | Fontil V [43] | Evaluation of a health information technology-enabled collective intelligence platform to improve diagnosis in primary care and urgent care settings: protocol for a pragmatic randomized controlled trial | JMIR Res Protoc | 2019 | Assist in the diagnostic process | Self-reported diagnostic confidence | USA | 12/15 | <https://doi.org/10.2196/13151> |
| 22 | Protocol | selfBACK | NCT03798288^e^ | Sandal LF [44] | An app-delivered self-management program for people with low back pain: protocol for the selfback randomized controlled trial | JMIR RES Protoc | 2019 | Low back pain | Pain-related disability at 3-month follow-up assessed using the RMDQ | Danish, Norwegian | 13/15 | <https://doi.org/10.2196/14720> |
| 23 | Protocol | DIAMANTE | NCT03490253 | Aguilera A [45] | MHealth app using machine learning to increase physical activity in diabetes and depression: clinical trial protocol for the diamante study | BMJ Open | 2020 | Diabetes and depression | Change in daily step counts | USA | 12/15 | <http://dx.doi.org/10.1136/bmjopen-2019-034723> |
| 24 | Protocol | EAGLE | NCT04000087^b^ | Yao X [46] | ECG AI-guided screening for low ejection fraction (EAGLE): rationale and design of a pragmatic cluster randomized trial | Am Heart J | 2020 | Low ejection fraction | The newly diagnosed left ventricular systolic dysfunction | USA | 9/15 | <https://doi.org/10.1016/j.ahj.2019.10.007> |
| 25 | Protocol | REINFORCE | NCT04473326 | Lauffenburger JC [47] | Reinforcement learning to improve non-adherence for diabetes treatments by optimising response and customising engagement (REINFORCE): study protocol of a pragmatic randomised trial | BMJ Open | 2021 | Diabetes | Average adherence to medication over the 6-month follow-up period | USA | 12/15 | <http://dx.doi.org/10.1136/bmjopen-2021-052091> |
| 26 | Protocol | Not available | ChiCTR2100053872 | Cheng W [48] | Evaluation of a village-based digital health kiosks program: a protocol for a cluster randomized clinical trial | Digit Health | 2022 | Village-based digital health | 1.Primary Care Utilization 2. Referrals to Upper-level facilities  3.Telemedicine Platform and Al Doctor Utilization | China | 8/15 | <https://doi.org/10.1177/20552076221129100> |
| 27 | Protocol | Not available | ISRCTN13817256^c^ | Chang Y [49] | Changing antibiotic prescribing practices in outpatient primary care settings in China: study protocol for a health information system-based cluster-randomised crossover controlled trial | PLOS One | 2022 | Antibiotic prescriptions | The 10-day antibiotic pre scription rate of physicians | China | 10/15 | <https://doi.org/10.1371/journal.pone.0259065> |
| 28 | Protocol | Not available | ISRCTN18106677 | Han JED [50] | Teleophthalmology-enabled and artificial intelligence-ready referral pathway for community optometry referrals of retinal disease (HERMES): a cluster randomised superiority trial with a linked diagnostic accuracy study-hermes study report 1-study protocol | BMJ Open | 2022 | Retinal disease | The proportion of false referrals | UK | 11/15 | <http://dx.doi.org/10.1136/bmjopen-2021-055845> |
| 29 | Protocol | Dcubed | NCT05231954 | Kleiman MJ [51] | Digital detection of dementia (D-3): a study protocol for a pragmatic cluster-randomized trial examining the application of patient-reported outcomes and passive clinical decision support systems | Trials | 2022 | Dementia | New Alzheimer’s disease and related dementias case identifed within 12 months | USA | 8/15 | <https://doi.org/10.1186/s13063-022-06809-5> |
| 30 | Protocol | Not available | ACTRN12621000174886 | Laranjo L [52] | Coordinating health care with artificial intelligence-supported technology for patients with atrial fibrillation: protocol for a randomized controlled trial | JMIR Res Protoc | 2022 | Atrial fibrillation | AF-related quality of life at 6 months | Australia | 11/15 | <https://doi.org/10.2196/34470> |
| 31 | Protocol | PROVIDENT | NCT05096429 | Marshall BDL [53] | Preventing overdose using information and data from the environment (PROVIDENT): protocol for a randomized, population-based, community intervention trial | Addiction | 2022 | Drug overdose | The 2-year cumulative incidence of fatal and non-fatal overdose at the city/town level | USA | 9/15 | <https://doi.org/10.1111/add.15731> |
| 32 | Protocol | Not available | ChiCTR2100052307 | Ru X [54] | Effect of an artificial intelligence-assisted tool on non-valvular atrial fibrillation anticoagulation management in primary care: protocol for a cluster randomized controlled trial | Trials | 2022 | Non-valvular atrial fibrillation anticoagulation management | The proportion of antithrombotic treatment prescriptions in agreement with recommendations in the latest China’s AF-related guidelines | China | 11/15 | <https://doi.org/10.1186/s13063-022-06250-8> |
| 33 | Protocol | Not available | Not available | Soto-Ruiz N [55] | Web-based personalized intervention to improve quality of life and self-efficacy of long-term breast cancer survivors: study protocol for a randomized controlled trial | Int J Environ Res Public Health | 2022 | Breast cancer | Quality of life | Spain | 9/15 | <https://doi.org/10.3390/ijerph191912240> |
| 34 | Protocol | HUA-TUO | NCT04523649 | Wong CK [56] | Protocol for home-based solution for remote atrial fibrillation screening to prevent recurrence stroke (HUA-TUO AF Trial): a randomised controlled trial | BMJ Open | 2022 | Atrial fibrillation/stroke | The time to first detection of AF at 2 years of follow-up | China | 11/15 | <https://doi.org/10.1136/bmjopen-2021-053466> |
| 35 | Protocol | Not available | NCT04673019 | Gordon BR [57] | Addressing metastatic individuals everyday: rationale and design of the nurse AMIE for Amazon Echo Show trial among metastatic breast cancer patients | Contemp Clin Trials Commun | 2023 | Metastatic breast cancer | Change in physical distress over three months | USA | 13/15 | <https://doi.org/10.1016/j.conctc.2023.101058> |
| 36 | Protocol | Not available | NCT04604457 | Heinzen EP [58] | Impact of a machine learning algorithm on time to palliative care in a primary care population: protocol for a stepped-wedge pragmatic randomized trial | BMC Palliat Care | 2023 | Palliative care | Time to record of consult in the electronic health record by the palliative care team in the outpatient setting | USA | 9/15 | <https://doi.org/10.1186/s12904-022-01113-0> |
| 37 | Protocol | SPIRO-AID | NCT05933694 | Doe G [59] | Comparing performance of primary care clinicians in the interpretation of SPIROmetry with or without Artificial Intelligence Decision support software (SPIRO-AID): a protocol for a randomised controlled trial | BMJ Open | 2024 | Spirometry | The performance of their preferred diagnosis | UK | 11/15 | <https://doi.org/10.1136/bmjopen-2024-086736> |
| 38 | Protocol | Dejal@ | NCT03445507^d^ | Avila-Tomas JF [60] | Effectiveness of a chat-bot for the adult population to quit smoking: protocol of a pragmatic clinical trial in primary care (Dejal@) | BMC Med Inform Decis Mak | 2019 | Quit smoking | Continuous tobacco abstinence at 6 months | Spain | 7/15 | <https://doi.org/10.1186/s12911-019-0972-z> |
| 39 | Protocol | Not available | NCT05486390 | Kwan YH [61] | Empowering patients with comorbid diabetes and hypertension through a multi-component intervention of mobile app, health coaching and shared decision-making: Protocol for an effectiveness-implementation of randomised controlled trial | PLoS One | 2024 | Comorbid diabetes and hypertension | Change in HbA1c level over 9 months | Singapore | 8/15 | <https://doi.org/10.1371/journal.pone.0296338> |
| 40 | Protocol | CHESS | NCT05605418 | Zhang H [62] | Design and rationale of the comprehensive intelligent Hypertension management system (CHESS) evaluation study: a cluster randomized controlled trial for hypertension management in primary care | Am Heart J | 2024 | Hypertension | The mean change from base line to 12-month follow-up in 24-hour ambulatory SBP measured by ABPM | China | 10/15 | <https://doi.org/10.1016/j.ahj.2024.03.018> |

^a^ Trial NCT03288142: Both the published RCT and its corresponding protocol were identified.

^b^ Trial NCT04000087: Both the published RCT and its corresponding protocol were identified.

^c^ Trial lSRCTN13817256: Both the published RCT and its corresponding protocol were identified.

^d^ Trial NCT03445507: Both the published RCT and its corresponding protocol were identified.

^e^ Trial lNCT03798288: Both the published RCT and its corresponding protocol were identified.

**Reference list**

1. Persell SD, Peprah YA, Lipiszko D, Lee JY, Li JJ, Ciolino JD, et al. Effect of home blood pressure monitoring via a smartphone hypertension coaching application or tracking application on adults with uncontrolled hypertension: a randomized clinical trial. JAMA Netw Open. 2020;3(3): e200255. [doi: 10.1001/jamanetworkopen.2020.0255] [Medline:32119093]

2. Seol HY, Shrestha P, Muth JF, Wi C-I, Sohn S, Ryu E, et al. Artificial intelligence-assisted clinical decision support for childhood asthma management: a randomized clinical trial. PLoS One. 2021;16(8): e0255261. [doi: 10.1371/journal.pone.0255261] [Medline: 34339438]

3. Yao X, Rushlow DR, Inselman JW, McCoy RG, Thacher TD, Behnken EM, et al. Artificial intelligence-enabled electrocardiograms for identification of patients with low ejection fraction: a pragmatic, randomized clinical trial. Nat Med. 2021;27(5):815-819. [doi: 10.1038/s41591-021-01335-4] [Medline: 33958795]

4. Itoh N, Mishima H, Yoshida Y, Yoshida M, Oka H, Matsudaira K. Evaluation of the effect of patient education and strengthening exercise therapy using a mobile messaging app on work productivity in Japanese patients with chronic low back pain: open-label, randomized, parallel-group trial. JMIR Mhealth Uhealth. 2022;10(5): e35867. [doi: 10.2196/35867] [Medline: 35576560]

5. Piette JD, Newman S, Krein SL, Marinec N, Chen J, Williams DA, et al. Patient-centered pain care using artificial intelligence and mobile health tools: a randomized comparative effectiveness trial. JAMA Intern Med. 2022;182(9):975-983. [doi: 10.1001/jamainternmed.2022.3178] [Medline: 35939288]

6. Rushlow DR, Croghan IT, Inselman JW, Thacher TD, Friedman PA, Yao X, et al. Clinician adoption of an artificial intelligence algorithm to detect left ventricular systolic dysfunction in primary care. Mayo Clin Proc. 2022;97(11):2076-2085. [doi: 10.1016/j.mayocp.2022.04.008] [Medline: 36333015]

7. Manz CR, Zhang Y, Chen K, Long Q, Small DS, Evans CN, et al. Long-term effect of machine learning-triggered behavioral nudges on serious illness conversations and end-of-life outcomes among patients with cancer: a randomized clinical trial. JAMA Oncol. 2023;9(3):414-418. [doi: 10.1001/jamaoncol.2022.6303] [Medline: 36633868]

8. Wei MT, Shankar U, Parvin R, Abbas SH, Chaudhary S, Friedlander Y, et al. Evaluation of computer-aided detection during colonoscopy in the community (AI-SEE): a multicenter randomized clinical trial. Am J Gastroenterol. 2023;118(10):1841-1847. [doi: 10.14309/ajg.0000000000002239] [Medline: 36892545]

9. Yang J, Cui Z, Liao X, He X, Wang L, Wei D, et al. Effects of a feedback intervention on antibiotic prescription control in primary care institutions based on a health information system: a cluster randomized cross-over controlled trial. J Glob Antimicrob Resist. 2023; 33:51-60. [doi: 10.1016/j.jgar.2023.02.006] [Medline: 36828121]

10. Lin CS, Liu WT, Tsai DJ, Lou YS, Chang CH, Lee CC, et al. AI-enabled electrocardiography alert intervention and all-cause mortality: a pragmatic randomized clinical trial. Nat Med. 2024;30(5):1461-1470. [doi: 10.1038/s41591-024-02961-4] [Medline: 38684860]

11. Benrimoh D, Whitmore K, Richard M, Golden G, Perlman K, Jalali S, et al. Artificial Intelligence in Depression - Medication Enhancement (AID-ME): a cluster randomized trial of a deep learning enabled clinical decision support system for personalized depression treatment selection and management. medRxiv. Preprint posted online on June 25, 2024.

12. Olano-Espinosa E, Avila-Tomas JF, Minue-Lorenzo C, Matilla-Pardo B, Serrano Serrano ME, Martinez-Suberviola FJ, et al. Dejal@ Group. Effectiveness of a conversational chatbot (Dejal@bot) for the adult population to quit smoking: pragmatic, multicenter, controlled, randomized clinical trial in primary care. JMIR Mhealth Uhealth. 2022;10(6): e34273. [doi: 10.2196/34273] [Medline: 35759328]

13. Sandal LF, Bach K, Øverås CK, Svendsen MJ, Dalager T, Stejnicher Drongstrup Jensen J, et al. Effectiveness of app-delivered, tailored self-management support for adults with lower back pain-related disability: a selfback randomized clinical trial. JAMA Intern Med. 2021;181(10):1288-1296. [doi: 10.1001/jamainternmed.2021.4097] [Medline: 34338710]

14. Goh E, Gallo R, Hom J, Strong E, Weng Y, Kerman H, et al. Large language model influence on diagnostic reasoning: a randomized clinical trial. JAMA Netw Open. 2024;7(10): e2440969. [doi: 10.1001/jamanetworkopen.2024.40969] [Medline: 39466245]

15. Granviken F, Meisingset I, Bach K, Bones AF, Simpson MR, Hill JC, et al. Personalised decision support in the management of patients with musculoskeletal pain in primary physiotherapy care: a cluster randomised controlled trial (the SupportPrim project). Pain. 2024. [doi: 10.1097/j.pain.0000000000003456] [Medline: 39432806]

16. Thiruvengadam NR, Solaimani P, Shrestha M, Buller S, Carson R, Reyes-Garcia B, et al. The efficacy of real-time computer-aided detection of colonic neoplasia in community practice: a pragmatic randomized controlled trial. Clin Gastroenterol Hepatol. 2024;22(11):2221-2230.e15. [doi: 10.1016/j.cgh.2024.02.021] [Medline: 38437999]

17. Nayak A, Vakili S, Nayak K, Nikolov M, Chiu M, Sosseinheimer P, et al. Use of voice-based conversational artificial intelligence for basal insulin prescription management among patients with type 2 diabetes: a randomized clinical trial. JAMA Netw Open. 2023;6(12): e2340232. [doi: 10.1001/jamanetworkopen.2023.40232] [Medline: 38039007]

18. Alcoceba-Herrero I, Coco-Martín MB, Jiménez-Pérez JM, Leal-Vega L, Martín-Gutiérrez A, Dueñas-Gutiérrez C, et al. Randomized controlled trial to assess the feasibility of a novel clinical decision support system based on the automatic generation of alerts through remote patient monitoring. J Clin Med. 2024;13(19):5974. [doi: 10.3390/jcm13195974] [Medline: 39408035]

19. Ortiz O, Daca-Alvarez M, Rivero-Sanchez L, Gimeno-Garcia AZ, Carrillo-Palau M, Alvarez V, et al. TIMELY study group. An artificial intelligence-assisted system versus white light endoscopy alone for adenoma detection in individuals with Lynch syndrome (TIMELY): an international, multicentre, randomised controlled trial. Lancet Gastroenterol Hepatol. 2024;9(9):802-810. [doi: 10.1016/S2468-1253(24)00187-0] [Medline: 39033774]

20. Persell SD, Karmali KN, Stein N, Li J, Peprah YA, Lipiszko D, et al. Design of a randomized controlled trial comparing a mobile phone-based hypertension health coaching application to home blood pressure monitoring alone: The Smart Hypertension Control Study. Contemp Clin Trials. 2018; 73:92-97. [doi: 10.1016/j.cct.2018.08.013] [Medline: 30172038]

21. Fontil V, Khoong EC, Hoskote M, Radcliffe K, Ratanawongsa N, Lyles CR, et al. Evaluation of a health information technology-enabled collective intelligence platform to improve diagnosis in primary care and urgent care settings: protocol for a pragmatic randomized controlled trial. JMIR Res Protoc. 2019;8(8):e13151. [doi: 10.2196/13151] [Medline: 31389337]

22. Sandal LF, Stochkendahl MJ, Svendsen MJ, Wood K, Øverås CK, Nordstoga AL, et al. An app-delivered self-management program for people with low back pain: protocol for the selfback randomized controlled trial. JMIR Res Protoc. 2019;8(12): e14720. [doi: 10.2196/14720] [Medline: 31793897]

23. Aguilera A, Figueroa CA, Hernandez-Ramos R, Sarkar U, Cemballi A, Gomez-Pathak L, et al. mHealth app using machine learning to increase physical activity in diabetes and depression: clinical trial protocol for the DIAMANTE Study. BMJ Open. 2020;10(8): e034723. [doi: 10.1136/bmjopen-2019-034723] [Medline: 32819981]

24. Yao X, McCoy RG, Friedman PA, Shah ND, Barry BA, Behnken EM, et al. ECG AI-guided screening for low ejection fraction (EAGLE): rationale and design of a pragmatic cluster randomized trial. Am Heart J. 2020; 219:31-36. [doi: 10.1016/j.ahj.2019.10.007] [Medline: 31710842]

25. Lauffenburger JC, Yom-Tov E, Keller PA, McDonnell ME, Bessette LG, Fontanet CP, et al. Reinforcement learning to improve non-adherence for diabetes treatments by optimising response and customising engagement (REINFORCE): study protocol of a pragmatic randomised trial. BMJ Open. 2021;11(12): e052091. [doi: 10.1136/bmjopen-2021-052091] [Medline: 34862289]

26. Cheng W, Zhang Z, Hoelzer S, Tang W, Liang Y, Du Y, et al. Evaluation of a village-based digital health kiosks program: a protocol for a cluster randomized clinical trial. Digit Health. 2022; 8:20552076221129100. [doi: 10.1177/20552076221129100] [Medline: 36211797]

27. Chang Y, Yao Y, Cui Z, Yang G, Li D, Wang L, et al. Changing antibiotic prescribing practices in outpatient primary care settings in China: study protocol for a health information system-based cluster-randomised crossover controlled trial. PLoS One. 2022;17(1):e0259065. [doi: 10.1371/journal.pone.0259065] [Medline: 34995279]

28. Han JED, Liu X, Bunce C, Douiri A, Vale L, Blandford A, et al. Teleophthalmology-enabled and artificial intelligence-ready referral pathway for community optometry referrals of retinal disease (HERMES): a cluster randomised superiority trial with a linked diagnostic accuracy study-hermes study report 1-study protocol. BMJ Open. 2022;12(2): e055845. [doi: 10.1136/bmjopen-2021-055845] [Medline: 35105593]

29. Kleiman MJ, Plewes AD, Owora A, Grout RW, Dexter PR, Fowler NR, et al. Digital detection of dementia (D): a study protocol for a pragmatic cluster-randomized trial examining the application of patient-reported outcomes and passive clinical decision support systems. Trials. 2022;23(1):868. [doi: 10.1186/s13063-022-06809-5] [Medline: 36221141]

30. Laranjo L, Shaw T, Trivedi R, Thomas S, Charlston E, Klimis H, et al. Coordinating health care with artificial intelligence-supported technology for patients with atrial fibrillation: protocol for a randomized controlled trial. JMIR Res Protoc. 2022;11(4):e34470. [doi: 10.2196/34470] [Medline: 35416784]

31. Marshall BDL, Alexander-Scott N, Yedinak JL, Hallowell BD, Goedel WC, Allen B, et al. Preventing overdose using information and data from the environment (PROVIDENT): protocol for a randomized, population-based, community intervention trial. Addiction. 2022;117(4):1152-1162. [doi: 10.1111/add.15731] [Medline: 34729851]

32. Ru X, Zhu L, Ma Y, Wang T, Pan Z. Effect of an artificial intelligence-assisted tool on non-valvular atrial fibrillation anticoagulation management in primary care: protocol for a cluster randomized controlled trial. Trials. 2022;23(1):316. [doi: 10.1186/s13063-022-06250-8] [Medline: 35428315]

33. Soto-Ruiz N, Escalada-Hernández P, Martín-Rodríguez LS, Ferraz-Torres M, García-Vivar C. Web-based personalized intervention to improve quality of life and self-efficacy of long-term breast cancer survivors: study protocol for a randomized

controlled trial. Int J Environ Res Public Health. 2022;19(19):12240. [doi: 10.3390/ijerph191912240] [Medline: 36231542]

34. Wong CK, Hai JJ, Lau YM, Zhou M, Lui HW, Lau KK, et al. Protocol for home-based solution for remote atrial fibrillation screening to prevent recurrence stroke (HUA-TUO AF Trial): a randomised controlled trial. BMJ Open. 2022;12(7):e053466. [doi: 10.1136/bmjopen-2021-053466] [Medline: 35840293]

35. Gordon BR, Qiu L, Doerksen SE, Kanski B, Lorenzo A, Truica CI, et al. Addressing metastatic individuals everyday: rationale and design of the nurse AMIE for Amazon Echo Show trial among metastatic breast cancer patients. Contemp Clin Trials Commun. 2023;32: 101058. [doi: 10.1016/j.conctc.2023.101058] [Medline: 36698743]

36. Heinzen EP, Wilson PM, Storlie CB, Demuth GO, Asai SW, Schaeferle GM, et al. Impact of a machine learning algorithm on time to palliative care in a primary care population: protocol for a stepped-wedge pragmatic randomized trial. BMC Palliat Care. 2023;22(1):9. [doi: 10.1186/s12904-022-01113-0] [Medline: 36737744]

37. Doe G, El-Emir E, Edwards GD, Topalovic M, Evans RA, Russell R, et al. Comparing performance of primary care clinicians in the interpretation of SPIROmetry with or without artificial intelligence decision support software (SPIRO-AID): a protocol for a randomised controlled trial. BMJ Open. 2024;14(6): e086736. [doi: 10.1136/bmjopen-2024-086736] [Medline: 38950987]

38. Avila-Tomas JF, Olano-Espinosa E, Minué-Lorenzo C, Martinez-Suberbiola FJ, Matilla-Pardo B, Serrano-Serrano ME, et al. Group Dej@lo. Effectiveness of a chat-bot for the adult population to quit smoking: protocol of a pragmatic clinical trial in primary care (Dejal@). BMC Med Inform Decis Mak. Dec 03, 2019;19(1):249. [doi: 10.1186/s12911-019-0972-z] [Medline: 31796061]

39. Kwan YH, Yoon S, Tai BC, Tan CS, Phang JK, Tan WB, et al. Empowering patients with comorbid diabetes and hypertension through a multi-component intervention of mobile app, health coaching and shared decision-making: protocol for an effectiveness-implementation of randomised controlled trial. PLoS One. 2024;19(2): e0296338. [doi: 10.1371/journal.pone.0296338] [Medline: 38408067]

40. Zhang H, Huo X, Ren L, Lu J, Li J, Zheng X, et al. Design and rationale of the Comprehensive Intelligent Hypertension Management System (CHESS) evaluation study: a cluster randomized controlled trial for hypertension management in primary care. Am Heart J. 2024; 273:90-101. [doi: 10.1016/j.ahj.2024.03.018] [Medline: 38575049]
