# Supplementary material for: Reporting Quality of AI Intervention in Randomized Controlled Trials in Primary Care: Systematic Review and Meta-Epidemiological Study
Source: J Med Internet Res. 2025 Feb 25;27:e56774. doi: 10.2196/56774 (PMC11897677; doi:10.2196/56774)
Supplement: Multimedia Appendix 4 [file jmir_v27i1e56774_app4.docx]

**Multimedia Appendix 4.** Association between the primary outcome result status in RCTs and the reporting quality of each item

| **Item** | | **Result of primary outcome, n (%)** | | ***P* value** |
| --- | --- | --- | --- | --- |
|  |  | **Positive** | **Negative** |  |
| **CONSORT-AI 1a(i) Elaboration** | |  |  | .23 |
|  | Reported | 8 (73) | 8 (100) |  |
|  | Not reported | 3 (27) | 0 (0) |  |
| **CONSORT-AI 1b(ii) Elaboration** | |  |  | >.99 |
|  | Reported | 11 (100) | 8 (100) |  |
|  | Not reported | 0 (0) | 0 (0) |  |
| **CONSORT-AI 2a (i) Extension** | |  |  | >.99 |
|  | Reported | 11 (100) | 8 (100) |  |
|  | Not reported | 0 (0) | 0 (0) |  |
| **CONSORT-AI 4a (i) Elaboration** | |  |  | >.99 |
|  | Reported | 10 (91) | 8 (100) |  |
|  | Not reported | 1 (9) | 0 (0) |  |
| **CONSORT-AI 4a (ii) Extension** | |  |  | >.99 |
|  | Reported | 3 (27) | 2 (25) |  |
|  | Not reported | 8 (73) | 6 (75) |  |
| **CONSORT-AI 4b Extension** | |  |  | >.99 |
|  | Reported | 11 (100) | 8 (100) |  |
|  | Not reported | 0 (0) | 0 (0) |  |
| **CONSORT-AI 5 (i) Extension** | |  |  | .38 |
|  | Reported | 8 (73) | 4 (50) |  |
|  | Not reported | 3 (27) | 4 (50) |  |
| **CONSORT-AI 5 (ii) Extension** | |  |  | .32 |
|  | Reported | 9 (82) | 4 (50) |  |
|  | Not reported | 2 (18) | 4 (50) |  |
| **CONSORT-AI 5 (iii) Extension** | |  |  | >.99 |
|  | Reported | 1 (9) | 1 (13) |  |
|  | Not reported | 10 (91) | 7 (83) |  |
| **CONSORT-AI 5 (iv) Extension** | |  |  | >.99 |
|  | Reported | 9 (82) | 6 (75) |  |
|  | Not reported | 2 (18) | 2 (25) |  |
| **CONSORT-AI 5 (v) Extension** | |  |  | .26 |
|  | Reported | 10 (91) | 5 (63) |  |
|  | Not reported | 1 (9) | 3 (38) |  |
| **CONSORT-AI 5 (vi) Extension** | |  |  | .60 |
|  | Reported | 9 (82) | 5 (63) |  |
|  | Not reported | 2 (18) | 3 (38) |  |
| **CONSORT-AI 19 Extension** | |  |  | .49 |
|  | Reported | 2 (18) | 0 (0) |  |
|  | Not reported | 9 (82) | 8 (100) |  |
| **CONSORT-AI 25 Extension** | |  |  | .23 |
|  | Reported | 3 (27) | 0 (0) |  |
|  | Not reported | 8 (73) | 8 (100) |  |
