# Supplementary material for: Reporting Quality of AI Intervention in Randomized Controlled Trials in Primary Care: Systematic Review and Meta-Epidemiological Study
Source: J Med Internet Res. 2025 Feb 25;27:e56774. doi: 10.2196/56774 (PMC11897677; doi:10.2196/56774)
Supplement: Multimedia Appendix 5 [file jmir_v27i1e56774_app5.docx]

**Multimedia Appendix 5.** Association between trial characteristics and the percentage of adequately reported items

| **Subgroup group** | | **Median (IQR) [Range]** | **No** | ***P* value^a^** | ***P* value for main effect^b^** | ***P* value for interaction effect^c^** |
| --- | --- | --- | --- | --- | --- | --- |
| **Year** | |  |  | .19 | .17 | .59 |
|  | 2023-2024 | 8.5 (7-9.25) [7-14] | 10 |  |  |  |
|  | Pre-2022 | 9 (8.5-10) [7-13] | 9 |  |  |  |
| **Blinding** | |  |  | .86 | .68 | .0502 |
|  | Yes | 9 (7-10) [7-14] | 7 |  |  |  |
|  | No | 9 (7.25-10) [7-13] | 12 |  |  |  |
| **Type of AI model** | |  |  | .40 | .49 | .86 |
|  | LLM | 9 (7.25-9) [7-10] | 8 |  |  |  |
|  | Others | 9 (7-10) [7-14] | 11 |  |  |  |
| **Deployment context** | |  |  | .08 | .09 | .72 |
|  | Clinician-assisted decision support | 9.5 (8.5-10.75) [7-14] | 10 |  |  |  |
|  | Others | 8 (7-9) [7-10] | 9 |  |  |  |
| **Primary outcomes by result status** | |  |  | .23 | Not applicable | Not applicable |
|  | Positive | 9 (7-10) [7-14] | 11 |  |  |  |
|  | Negative | 8.5 (7.25-9) [7-10] | 8 |  |  |  |
| **Type of disease** | |  |  | .02 | .03 | .39 |
|  | Cardiovascular disease | 11.5 (9.25-13.75) [9-14] | 4 |  |  |  |
|  | Others | 9 (7-9) [7-10] | 15 |  |  |  |

^a^ The percentage of adequately reported items was compared using the Mann-Whitney U test.

^b^ *P* value for the main effects between primary outcome result status and various RCT characteristics on the percentage of adequately reported items using the Scheirer-Ray-Hare test.

^c^ *P* value for the interaction effects between primary outcome result status and various RCT characteristics on the percentage of adequately reported items using the Scheirer-Ray-Hare test.
